# Seborrheic dermatitis due to *Malassezia* species in Ahvaz, Iran

**Published:** 2013-09

**Authors:** Ali Zarei-Mahmoudabadi, Majid Zarrin, Forough Mehdinezhad

**Affiliations:** 1 Department of Medical Mycology, School of Medicine, Ahvaz Jundishapur University of Medical Sciences, Ahvaz, Iran; 2 Infectious and Tropical Diseases Research Centre, Ahvaz Jundishapur University of Medical Sciences, Ahvaz, Iran

**Keywords:** Seborrheic dermatitis, Pityriasis versicolor, *Malassezia*, *M. globosa*

## Abstract

**Background and Objective:**

Seborrheic dermatitis (SD) is a frequent disorder of the skin that is distinguished by the development of erythematous patches and yellow-gray scales. It is a multifactor disease that requires predisposing factors for its progress. Presence of these factors leads to reproduction of opportunistic yeast *Malassezia* spp. The aim of the present study was to isolate and identify distribution of *Malassezia* species on the scalp of SD patients in Ahvaz using modified Dixons agar.

**Materials and Methods:**

A total of 110 patients diagnosed with SD were sampled. The sampling was carried out by brushing the hair and collecting the dandruff in paper pockets. For identification of *Malassezia* species, the scalp scales were cultured in Dixons agar. A combination of different characteristics including yeast cell morphology, ability to grow on Sabouraud dextrose agar, catalase test and ability to utilize individual Tweens (20, 40, 60 & 80) were used for identification of species.

**Results:**

Twenty-seven of 110 (24.5%) SD patients had positive cultures for *Malassezia* species of which 17 (63%) were male and 10 (37%) were female. The most commonly identified *Malassezia* species was *M. globosa* (40.7%) followed by *M. pachydermatis* (22.2%), *M. furfur* (11.1%) and *M. restricta*(7.4%) and *Malassezia* species (18.5%).

**Conclusion:**

*Malassezia globosa* was considered to be the most important *orgaism involved in cases* with Seborrheic dermatitisin this study.

## INTRODUCTION

Superficial mycoses are fungal infections that invade keratinized layer of skin and hair shafts of the human body. These infections usually are asymptomatic and without cellular and/or humoral responses. Pityriasis versicolor is a chronic and mild superficial mycosis caused by several species of *Malassezia*, a human normal flora. Pityriasis versicolor has worldwide distribution; however it is more prevalent in tropical and subtropical areas. Several reports showed that its prevalence is 20%-50% in tropical areas (1–3). Disease is more common in young people with age range 20-30 year, however it is not uncommon in children in tropics (4). Pityriasis versicolor is one of the most common fungal infections in Iran, especially in north and south, south west of the country that have tropical conditions (5–8). In addition, chronic infections, corticosteroid therapy, pregnancy and genetically factors have important role in disease.

*Malassezia* species are associated with pityriasis versicolor, folliculitis, Seborrheic dermatitis (SD), and atopic dermatitis (9, 10). In addition, *Malassezia* colonize healthy skin in human and some animals. Seborrheic dermatitis (SD) is a frequent disorder of the skin that is distinguished by the development of erythematous patches and yellow-gray scales. Seborrheic dermatitis is a multifactor disease that requires predisposing factors for its progress. Presence of these factors leads to reproduction of opportunistic yeast *Malassezia* spp.

Seborrheic dermatitis is usually occurring in young adults and disease is most often seen on the areas of the body rich in sebaceous glands (face, scalp, upper trunk) (11, 12). Dandruff and SD are associated with the presence of sebum, *Malassezia* metabolism, and individual susceptibility (13). The distribution disease is overall 1-5% suggested and can affect any ethnicity (12). However disease is more prevalent in male than female.

Most of the *Malassezia* species are lipophilic organisms and are part of human normal flora, especially greasy (oily) skin. Currently more than 14 species of *Malassezia* detected as causative agents of pityriasis versicolor, however the most common agents are *M. globosa*, *M. furfur, M. obtusa*, and *M*. *sympodialis* (14–16). Other agents are as follows; *M. restricta*, *M. slooffiae*, *M. pachydermatis*, *M. dermatis*, *M. japonica*, *M. nana*, *M. yamatoensis*, *M. equina*, *M. caprae*, and *M. cuniculi* (4, 11, 16). The aim of the present study was to isolate and identify *Malassezia* species from SD using modified Dixons agar in the university students of medical sciences in Ahvaz.

## MATERIALS AND METHODS

### Modified Dixons agar

Modified Dixons agar medium was prepared based on Shams et al. (17); 3.6% malt extract (Merck, Germany), 1.2% agar (Merck, Germany), 2% bile salts (Sigma, UK), 1% Tween 40 (Merck, Germany), 0.2% glycerol (Merck, Germany), 0.2% oleic acid (Merck, Germany), supplemented with cycloheximide (0.5%) and chloramphenicol (0.05%) were sterilized at 121°C. Then, 0.6% filtered L-tryptophan (Sigma, USA) were added and divided into test tubes and cooled as slants.

### Sampling and culture

In the present study 110 medical students diagnosed with SD (not simple dandruff) (64 male and 46 female) were sampled. Age, gender and disease extent for each student were recorded. Scalp scales were collected in sterile sampling pockets using hairs brushing. Samples were transferred to the medical mycology laboratory, Ahvaz Jundishapur University of Medical Sciences. Scalp scales were cultured in two sets of Dixons agar tubes and incubated at 37°C for two weeks aerobically and considered every 2-3 days for growing.

### Isolation and identification procedures

Cultured tubes were examined every two days for growth. The *Malassezia* isolates were detected by its morphological and biochemical and physiological characteristics. For each positive sample several diagnostic tests including grow on the medium without lipid supplementation (Sabouraud dextrose agar, SDA (Merck, Germany), lipid-dependent species by the Tween assimilation method (Tween 20, 40, 60 and 80) and catalase reaction (2, 10, 17).

### Production of brown colony on Dixon agar

Deep brown pigmentation by *M. furfur* was observed on Dixons agar with tryptophan, whereas other species produced no pigmentation in medium (17).

### Cell morphology

A direct smear from grown colonies in Dixon agar after 10-14 days at 37°C was prepared. Yeast cell morphology was studied by methylene blue stained smears.

### Growth on SDA

Sabouraud dextrose agar slants inoculated with a portion of colony of *Malassezia* grown on Dixons agar and incubated at 37°C for two weeks. Grow on SDA medium was assessed by the presence of white-cream and brittle colonies of *Malassezia*.

### Catalase reaction

A drop of hydrogen peroxide (H_2_O_2_) was put on a portion of a colony on a clean glass slide and the production of gas bubbles indicated a positive reaction for catalase.

### Ability to utilize Tween

A suspension of each *Malassezia* species in sterile distilled water was prepared and adjusted to about 10^5^ cell/ml. Two milliliter of suspension was added into 16 ml melted sterile SDA and vigorously mixed and poured into a 9 cm diameter petri dish. Plates were put at ambient temperature for solidification. Then four wells with 2 mm diameter punch in each plates and filled with 5µl of sterile Tweens 20, 40, 60 and 80 respectively. Plates were incubated at 32°C for one week. The degree of growth (precipitation) around each well indicates utilization of Tween by *Malassezia* (2, 17).

## RESULTS

In the present study, 110 students diagnosed with SD (64, 58.2% male; 46, 41.8% female) were sampled. The age range of subjects is shown in Table 1. As shown in Table 1, the age range of 65.5% of subjects was 21-25 year. The duration disease in subjects were classified in three groups, six month in 12 (10.9%), 6-12 month in 25 (22.7%) and more than one year in 73 (66.4%). In the present study a total of 27 (24.5%) patients had positive cultures for *Malassezia* species that 17 (63%) were male and 10 (37%) female. Based on morphological, biochemical and physiological tests, 22 (81.5%) of positive cultures were detected into four species. *M. globosa* was the most commonly isolated species in subjects (11, 40.7%) followed by *M. pachydermatis* (6, 22.2%), *M. furfur* (3, 11.1%), *M. restricta* (2, 7.4%) and five isolates were unidentified (*Malassezia* species, 5, 18.5%).

**Table 1:** Age range sampled seborrheic dermatitis patients.

Age range	Male	Female	Total
<20	13(11.8%)	12(10.9%)	25(22.7%)
21-25	42(38.2%)	30(27.3%)	72(65.5%)
26-30	6(5.5%)	4(3.6%)	10(9.1%)
>30	3(2.7%)	0(0.0%)	3(2.7%)
Total	64(58.2%)	46(41.8%)	110(100%)

## DISCUSSION

Although the exceptional cause of SD is unknown, increase of *Malassezia* population in skin has been described as an important contributing factor (12). All *Malassezia* species (exception *M. pachydermatis*) are able to degrade lipids in sebum, consume certain saturated fatty acids and produce free fatty acids and triglycerides. Several studies have shown that SD is a chronic disease that affects adults, adolescents and infants (12, 18, 19). Many investigators and clinicians believe that *Malassezia* spp play an important role in the pathogenesis of SD (20, 21).

The incidence of SD in general population is approximately 11.6%, whereas this rate in infants in the first three months of life is 70% (22). In a study from Iran conducted by Zarrin *et al*. (8) SD was considered as the common superficial fungal disease among primary school students in Ahvaz. In our study only 24.5% of examined patients had positive culture for *Malassezia* species. It is indicated that another factors associated with SD in our patients and *Malassezia* species have few role in SD in this area. However, Lee *et al*. (23) report showed that *Malassezia* species play an important role in the pathogenesis of Korean SD patients and 85% of them contaminated to *Malassezia* species. In a study conducted by Hedayati *et al*. (24), 77% of the SD specimens were yielded *Malassezia* in north of Iran. The rate of recovery of *Malassezia* organisms from SD patients is higher in this study compare with our result, probably because of different climate in north of Iran which is very humid.

Del Rosso (12) in a report from the USA believes that most cases of SD appear in men than women and this rate was 1.7 in our study. In addition, the most cases of SD with positive culture were distributed at age range 21-25 years old. Several reports showed that the rate of *Malassezia* species in the twenties with SD is high because of sebaceous glands greatest activity (24, 25). Seborrheic dermatitis is a chronic disease and some cases may be more persistent with recurrences over several months (12). Our study shows that 66.4% of patients had duration more than one year whereas 10.9% affected less than six months.

Different *Malassezia* species were reported as causative agents of SD in the different countries. Lee *et al*. (23) reported *M. restricta*as the most important species in Korean SD patients. In addition, Prohic (26) in a study from Bosnia and Herzegovina believes that *M. restricta* (27.5%) is the main agents of SD and *M. globosa* (17.5%) and *M. slooffiae* (15%) are the next agents. In a molecular study by Tajima *et al*. (11), *M. restricta* and *M. globosa* were detected as the predominate agents of SD. In contrast, in Hedayati *et al*. study in north of Iran *M. globosa* was reported as the most frequently agent on scalp and face lesions, whereas *M. furfur* had most frequency on trunk lesions (24). In the present study, out of the 110 scalp scales that were cultured on Dixons agar, 24.5% yielded *Malassezia* that the most frequently *Malassezia* species was *M. globosa* (40.7%), followed by *M. pachydermatis* (22.2%), *M. furfur* (11.1%) and *M. restricta* (7.4%).

In conclusion, the results of present study showed low recovery rate (24.5%) of *Malassezia* species on patients with SD. In addition *Malassezia globosa* is considered to be the most important *Malassezia* species in our SD patients.
